# First case report of an adrenocortical carcinoma caused by a *BRCA2* mutation

**DOI:** 10.1097/MD.0000000000004756

**Published:** 2016-09-09

**Authors:** Nada El Ghorayeb, Solange Grunenwald, Serge Nolet, Vanessa Primeau, Stéphanie Côté, Christine M. Maugard, André Lacroix, Louis Gaboury, Isabelle Bourdeau

**Affiliations:** a Division of Endocrinology; b Department of Pathology; c Division of Clinical Genetics, Department of Medicine and Research Center (CRCHUM), Centre hospitalier de l’Université de Montréal, Montreal, QC, Canada.

**Keywords:** adrenocortical carcinoma, *BRCA2*, *TP53*

## Abstract

**Background:**

Adrenocortical carcinoma (ACC) may rarely be a component of inherited cancer syndromes such as Li-Fraumeni syndrome and Beckwith-Wiedemann syndrome. ACC caused by a *BRCA2* mutation has never been reported.

**Methods:**

Nucleotide sequencing of *BRCA2* in lymphocyte and tumoral DNA of a 50-year-old male who presented with an androgen-secreting ACC and a strong family history of breast, ovarian, and pancreatic cancers.

**Results:**

A germline *BRCA2* 2 bp heterozygous deletion at nucleotide 8765 (8765delAG) leading to a frameshift mutation (p.Glu2846GlyfsX23) was detected. Only the *BRCA2* deleted allele was retained in the ACC tumoral DNA compared with the control DNA supporting a loss of heterozygosity in the tumor.

**Conclusion:**

This is the first reported case of a patient with ACC associated with a *BRCA2* germline mutation. Loss of heterozygosity in ACC DNA suggests a causal link with the *BRCA2* 8765delAG mutation.

## 1 Introduction

Adrenocortical carcinomas (ACC) are mainly sporadic, but may be found in 3% to 5% of some genetic syndromes such as Li-Fraumeni syndrome (LFS), Beckwith-Wiedemann syndrome (BWS), Lynch syndrome, familial adenomatous polyposis (FAP), and in <1% of multiple endocrine neoplasia type 1 (MEN1) syndromes.^[1,2]^ Germline mutations in *BRCA1* (MIM 113705) and *BRCA2* (MIM 600185) genes account for cancer predisposition in majority of families with breast only or breast–ovarian cancer families.^[3]^ To date, *BRCA2* mutation in ACC has never been reported. We describe here a case of ACC associated with a germline *BRCA2* mutation in a family whose cancer history was compatible with a Li-Fraumeni-like (LFL) syndrome.

### 1.1 Clinical case

**Figure 1:**
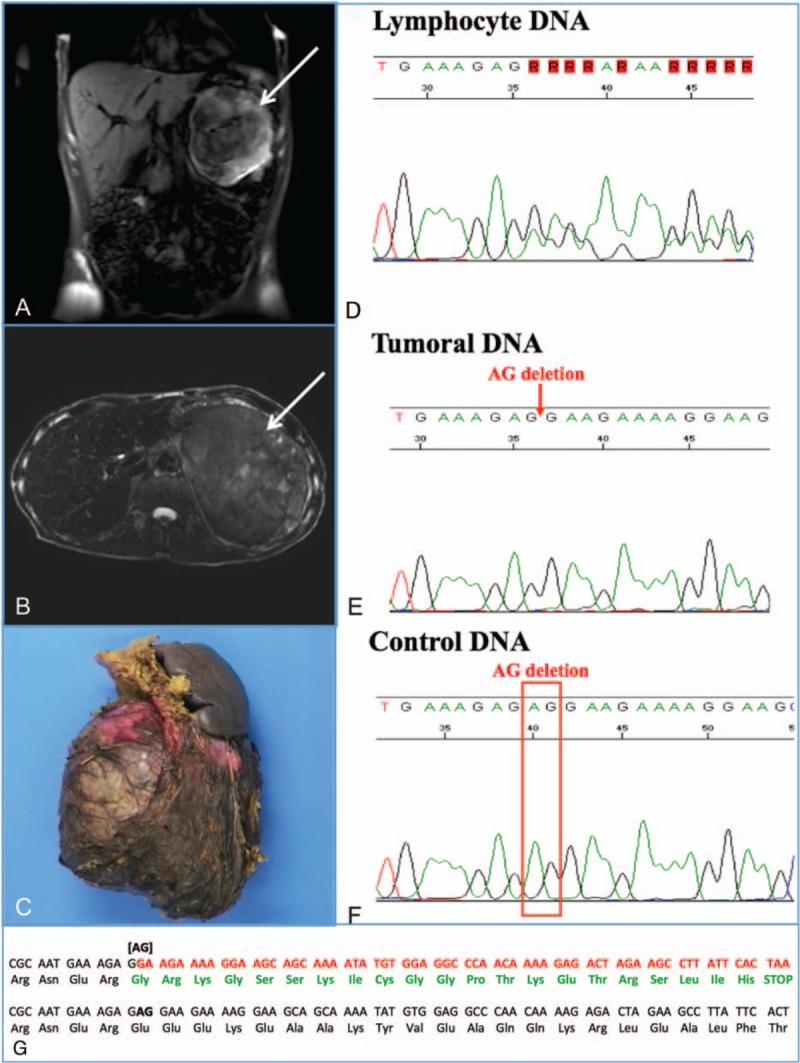
Abdominal MRI in sagittal (A) and transverse (B) sections of the patient showing an 18.8 × 11 × 14.9 cm mass in the left anterior pararenal space with a hyperintense signal in T1 and several hypointense nodules. (C) ACC, macroscopic picture. Nucleotide sequencing of *BRCA2* gene exon 20 revealed a 2 bp heterozygous deletion 8765delAG or c.8537_8538delAG leading to a frameshift (p.Glu2846Glyfs) and a stop codon in the leucocyte DNA from the patient (D). Only the deleted allele was retained in the ACC tumoral DNA (E) compared with a nonmutated control DNA (F), suggesting a loss of heterozygosity in the tumor. (G) The frame shift changes on the amino acid sequences (amino acids in green) compared with the normal amino acid sequences (amino acids in black).

A 50-year-old French Canadian male with no previous medical history was evaluated for a palpable lesion in his left flank. He denied abdominal pain; his review of systems and physical examination were only pertinent for the palpable mass that was hard and nontender. Ultrasound and MRI showed a retroperitoneal mass of 18.8 × 11 × 14.9 cm (Fig. 1A, B) suspicious of an adrenal tumor. The endocrine work-up was negative except for increased levels of DHEA-S (9.9 μmol/L N: 0.5–5.5). Following surgical resection, pathology confirmed an ACC of 18 × 17 × 13 cm with negative margins (Weiss score >3) (Fig. 1C). The patient was referred to us postoperatively with a unique lytic lesion of the right acetabulum present on fluorodeoxyglucose positron emission tomography (FDG-PET) scan. Bone CT scan and scintigraphy suggested a bone metastasis, which was confirmed by biopsy, consequently establishing a diagnosis of stage IV ACC.

**Figure 2:**
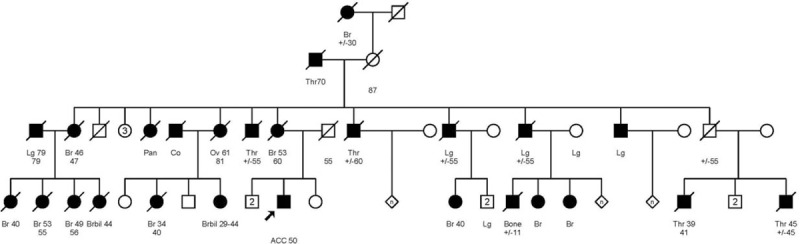
Familial pedigree of the patient's maternal branch, in which the French Canadian founder mutation *BRCA2* 8765delAG was found to be associated with a predisposition for breast, ovarian, and pancreatic cancers. The arrow shows the index patient. Squares: men, circles: women, black figures: individuals with cancers. Diagonal line = deceased person. Ages upper levels: age at diagnosis, ages lower levels: age of death. Br = breast cancer, Brbil = bilateral breast cancer, Bone = osteosarcoma, Co = colon cancer, Lg = lung cancer, Ov = ovarian cancer, Thr = throat cancer, Pan = pancreatic cancer.

The patient was offered genetic counseling; his family history revealed that his mother was diagnosed with breast cancer at 53 years old (yo). Among his maternal aunts, one developed breast cancer at 46 yo, one was affected by ovarian cancer at 61 yo, and another one had pancreatic cancer (Fig. 2). Nine female maternal cousins were affected with breast cancer between 29 and 53 yo, 2 of them were early-onset breast cancers diagnosed before the age of 35. Another cousin was suspected to be affected by an osteosarcoma at 11 yo (Fig. 2). The 8765delAG *BRCA2* mutation was identified in this family.

### 1.2 Molecular genetic analysis

After giving his written informed consent, the patient had genetic analysis for the 8765delAG *BRCA2* mutation and the *TP53* gene. Exon numbering is based on the NCBI references sequences U43746 and NC_000017.9, respectively. Lymphocyte DNA was obtained and tumoral DNA was extracted from microdissected formalin-fixed paraffin embedded ACC tumor after a 56°C overnight Proteinase K digestion in an extraction buffer (50 mM Tris–HCl, pH 7.5; 1 mM EDTA; 0.5% Tween 20, 1 mg/mL Proteinase K) using a laboratory-developed method. *BRCA2* exon 20 was amplified by a polymerase chain reaction and directly sequenced (Applied Biosystems, Foster City, California, USA). *TP53* gene was analyzed by multiplex ligation-dependent probe amplification and direct sequencing. The germline *BRCA2* 2 bp heterozygous deletion at nucleotide 8765 (8765delAG) (Breast Cancer Information Core nomenclature) or c.8537_8538delAG (human genome variation society nomenclature) was identified. The deletion leads to a frameshift mutation (p.Glu2846Glyfs) and a stop codon (Fig. 1D, G). Only the *BRCA2* deleted allele was retained in the ACC tumoral DNA compared with the control DNA suggesting a loss of heterozygosity in the tumor (Fig. 1E–G). In addition, *TP53* gene analysis revealed a previously reported heterozygous polymorphism in exon 4 (c.215C>G, p.Pro72Arg) (rs1042522, NM_000546.5).

### 1.3 Interventions and outcome

Surgical resection of the ACC metastasis was followed by irradiation. Mitotane therapy was initiated, and it was well tolerated with no serious side effects with doses escalating progressively up to 6 g/d. He was replaced by hydrocortisone (60 mg/d) for adrenal insufficiency. Unfortunately 9 months later, 2 new hepatic lesions and 3 pulmonary nodules were identified by FDG-PET scan. He received several regimens of chemotherapy: 3 cycles of EDP (etoposide, doxorubicin and cisplatin), and then Streptozocin followed by sunitinib with no serious adverse events related to therapy. Unfortunately, therapeutic failure was evident by disease progression, so he received palliative care until he passed away 3 years after his initial diagnosis and surgery.

## 2 Discussion

ACC is rare with an incidence of 0.7 to 2.0 cases per million populations per year.^[4]^ Analyses of inherited syndromes related to ACC led to the progress in the pathogenesis of ACC including the LFS due to germline *TP53* mutations, the BWS due to the deregulation of imprinted genes in the chromosome 11p15.5 region, which contains the insulin-like growth factor 2 and the closed linked *H19* gene in the imprinting center 1, the Lynch syndrome due to a defect in the mismatch repair system (*MSH2, MSH6, MLH1*), and more rarely the MEN1 (*menin*) and FAP syndromes (*APC*).^[1,2]^

LFS results from a germline mutation in the *TP53* gene predisposing individuals to cancers with early onset, including breast cancer, soft tissue and osteosarcomas, brain cancers, leukemia, and ACC. TP53 mutations account for up to 50% to 70% of the families with classic LFS.^[5]^ Patients presenting with incomplete features of LFS are referred as having LFL syndrome. Up to 20% to 40% of LFL families carry TP53 mutations.^[5]^ Of all the cancers associated with mutations of *TP53*, ACC is the one whose frequency is the most increased compared with the general population.^[6]^ The family history of our patient prompted us to search for a genetic cause of his ACC. Two genes were studied: *BRCA2* because the French Canadian founder mutation 8765delAG was previously found to be associated with breast, ovarian, and pancreatic cancers in his maternal family, and *TP53* because of his family history and his diagnosis of ACC; recently the prevalence of germline *TP53* mutations in apparently sporadic adult ACC patients was found to be between 3% and 6%^[7,8]^ supporting to propose *TP53* genetic analysis to all patients with ACC.

We found that our patient was a heterozygous carrier of the 8765delAG *BRCA2* mutation. The *BRCA2* 8765delAG or c.8537_8538delAG mutation was first described in breast cancer families from French-Canadian and Jewish-Yemenite populations^[9,10]^ and is one of the most frequent founder mutations reported in French Canadian breast-only or breast-ovarian cancer families; many of the families had members with cancers at other sites but no ACC was described in *BRCA2*-linked families to this date.^[11]^ Two cases of adrenal tumors in *BRCA1/BRCA2* mutation carriers have been described previously: the first was a cystic lymphangioma in a 46 yo Ashkenazi woman diagnosed with breast and ovarian cancer and carrying the 185delAG mutation in the *BRCA1* gene; the second was a pheochromocytoma in a 61 yo Ashkenazi woman diagnosed with breast cancer and harboring the *BRCA2* 6174delT mutation. Both masses were removed, but no tumoral DNA was available for genetic studies.^[12]^ Adrenal tumors in these cases could have occurred by coincidence. In the case of our patient, loss of heterozygosity in ACC tumoral DNA as described previously in most tumors related to *BRCA2* gene mutations^[13]^ suggests a causal link between the *BRCA2* 8765delAG mutation and the ACC. The simultaneous presence of *TP53* gene p.Pro72Arg polymorphism and the *BRCA2* 8765delAG mutation may suggest a potential interaction between these 2 genetic defects.

This case of ACC suggests that a detailed medical and family history may reveal an unsuspected underlying hereditary condition and should be performed in all patients with ACC. Patients should be referred for specialized genetic counseling to understand the risks and benefits of genetic testing; adequate informed consent should be obtained as well as posttest genetic counseling. Based on recent findings, in the absence of family history suggesting any other genetic conditions, at least the *TP53* gene analysis should be offered to all ACC patients.^[7,8]^ Furthermore, the efficacy of ACC surveillance strategy among children found to have a *TP53* mutation was proven with better outcome and survival.^[14]^ Thus, the identification of an unsuspected germline *TP53* mutation may entail a clinical surveillance protocol for the detection of asymptomatic nonadrenal neoplasms in individuals with germline *TP53* mutations.^[15]^

## 3 Conclusions

This is the first reported case of ACC associated with a *BRCA2* germline mutation. Loss of heterozygosity in tumoral DNA confirms that the *BRCA2* 8765delAG mutation plays a role in adrenal oncogenesis supporting that ACC may be included in the spectrum of cancer-related *BRCA2* gene.

## Acknowledgments

The authors thank the patient and the patient's family, and also thank Christine Caron, Laurence Buisseret, John Stagg, and Katia Kaceres for technical assistance.
